# Supplementary material for: Near-atomistic simulations reveal the molecular principles that control chromatin structure and phase separation
Source: Nat Commun. 2026 Oct 3;17:10532. doi: 10.1038/s41467-026-78050-6 (PMC13642264; doi:10.1038/s41467-026-78050-6)
Supplement: Supplementary file 2 [file 41467_2026_78050_MOESM2_ESM.pdf]

nature portfolio

Peer Review File

Near-atomistic simulations reveal the molecular principles that control chromatin structure and phase separation

Corresponding Author: Professor Rosana Collepardo

This file contains all reviewer reports in order by version, followed by all author rebuttals in order by version.

Version 0:

Reviewer comments:

Reviewer #1

(Remarks to the Author)
This MD study represents a major advance in the simulation of larger system of nucleosome fibers with residue resolution. A crucial step to reach relevant system sizes is to replace the rigid basepair representation of the DNA model by a fully particle based description. This leads to a speed-up of about one order of magnitude, for the first time allowing to connect residue level details to reasonably large scales. The model has been reparametrized compared to the slower model and is highly predictive (e.g. sedimentation coefficients, condensate formation). This leads to exciting insights and promises to be of great importance also for future studies. For instance, Fig. 3 reports how the contacts of histone tails to other nucleosomes depend on the DNA linker length. Access to this kind of information is crucial as it allows to predict and interpret a wide range of physical properties and associated biological functions of chromatin. The section on histone tail acetylations is a concrete proof of this, as it is demonstrates that acetylations which have a strong effect on interactions in the model correspond to residues that also play an important role as acetylation marks in the biological system. I strongly recommend publication of this well-written manuscript after the following points (all minor) have been considered.

1. I noticed that figures in the main text are not always referred to. For instance, Fig. 3c and d do not appear to have been mentioned in the main text. In my opinion, any part of a figure which is not used in the main text belongs to supplementary material. Please check throughout the manuscript.

2. When arriving at the conclusion and reading about the differences with respect to histone tail conformations between 10N and 10N+5 linkers, I had the impression, that I had read about this point already several times before in this manuscript. Please check whether this is really the case and if so, please try to shorten or remove repetitions. But please leave the text in the discussion untouched, as it is well written and provides an excellent summary.

2. line 112: when referring to near-atomistic coarse grained models, please also cite work by Giovanni Brandani & Shoji Takada [I am not one of the authors].

typos: line 182: “represented represented”; line 604: earleir

line 604: do you really mean Fig. 5a?

FigS1: figure has parts a and b but legend does not refer to this.

Finally, this new approach allows for many exciting projects in the future. My “wish list” for future projects would include that the authors consider pulling on single chromatin fibers, isolated or immersed in a condensate, and to figure out the different contributions (DNA elasticity, histone tails …) to the force-extension curves for different linker lengths. Also I would consider it highly interesting if the authors manage to allow for more extended nucleosome breathing in their model (not just 10 bps from each end) and then study how the breathing of individual nucleosomes is affected by being part of a chromatin fiber/condensate.

(Remarks on code availability)

Reviewer #2

(Remarks to the Author)
This manuscript presents an exciting advance in the field of chromatin modeling and simulation by introducing a GPU-accelerated, OpenMM-based, near-atomistic (residue-resolution) chromatin model that was reparameterized from the authors' earlier work implemented in LAMMPS on CPUs [25]. The model has been applied to simulate a large chromatin system comprising 108 nucleosomes and to study liquid-liquid phase separation of tetranucleosomes with different linker lengths, presenting a compelling demonstration of the model's application to large chromatin systems with atomistic accuracy and providing insights into the role of disordered chromatin regions in phase separation.

I have several suggestions and questions regarding the validation and parameterization of this model.

1. The authors imposed 10 base pairs of nucleosomal DNA unwrapping in their model. This small degree of nucleosome unwrapping incurs close to zero free energy penalty (see Forties et al., 2011, Nucleic Acids Res) and therefore reflects the "unbreathing" mode described in the authors' earlier paper [25]. More rationale needs to be provided for this choice, as [25] determines that nucleosome breathing is critical for nucleosome interactions, especially in phase separation.
2. The manuscript shows excellent reproduction of the chromatin compaction difference between 10N and 10N+5 linker DNA, with numbers almost quantitatively reproducing the experimental results. However, the authors seem to suggest this arises from their incorporation of the CGeNArate model, a sequence-dependent DNA model. The differences in chromatin structure at different linker DNA lengths more likely arise from geometry, as analyzed in this paper, which appears to somewhat contradict the sequence-specific effect. Can the authors reconcile these observations?
3. Similarly, in Figure S3, the authors show that their original LAMMPS model is unable to reproduce the different chromatin structures at 10N and 10N+5 linker DNA, but was this performed in unbreathing mode? If so, would allowing more nucleosomal DNA unwrapping enable the model to reproduce the trend?
4. Similarly, would testing sequence-dependent chromatin properties provide a more direct validation of the model's advancement in incorporating DNA sequence specificity? For example, Cui et al. (PNAS, 2000) measured nucleosome pulling forces on chicken erythrocyte chromatin fiber, while Kruithof et al. (Nat. Struct. Mol. Biol., 2009) measured forces on pulling 601-sequence chromatin. Validation of these or other sequence-dependent chromatin properties by this model would strengthen its applicability for studying DNA sequence specificity.
5. One of the highlights is that the model uses a relatively small number of parameters (Table S1) while being able to reproduce a large number of structural properties of chromatin. To achieve this, the authors used a grid search to find the optimal parameters. Would it be beneficial to also show results at 5 mM NaCl, the lowest salt concentration, and demonstrate that the model can fully reproduce the fully expanded chromatin at very low salt concentration?
6. In Figure 3, the authors could consider showing intra-nucleosome versus inter-nucleosome contact frequencies. This would strengthen the authors' later analyses of those interactions at different linker DNA lengths.

Minor:
1. Can the authors explain the overall smaller sedimentation coefficient (more expanded chromatin) from simulations at 60 mM compared to the experimental results?
2. The simulations reported in Figure 7 could be extended so that larger portions of the simulation trajectories are included in the analysis.
3. The final 16x benchmark report on the 12-nucleosome fiber in Figure 1 is noteworthy and is often overlooked within the computational community. The authors could also report computer memory usage, which would provide helpful guidance for selecting appropriate GPU types for these simulations.
4. Some typos, such as IIA: "which represented represented histones at residue resolution and DNA at the base-pair-step level using ellipsoids"

(Remarks on code availability)
The authors have provided the necessary codes to reproduce the results presented in this manuscript. I did not run through their examples directly. I suggest that the authors could include more detailed instructions on how to execute these codes, specifying the input and expected output for each code. Additionally, it would be helpful if the codes were organized according to the results reported in the manuscript.

Reviewer #3

(Remarks to the Author)
Dear Editor and Authors,

This manuscript presents OpenCGChromatin, a near-atomistic coarse-grained chromatin framework that substantially extends chemically specific simulations into the regime of hundreds of nucleosomes and gene-length DNA, while retaining residue/nucleotide-level interpretability. The work is timely and impactful: it combines (i) strong validation against established linker-length–dependent compaction/structural benchmarks, (ii) direct-coexistence simulations to interrogate chromatin phase behavior, and (iii) a clear mechanistic narrative linking linker geometry to nucleosome stacking frustration, histone-tail exposure, multivalency/network connectivity, and condensate formation. Overall, I find the manuscript strong and well executed.

Recommendation: Accept pending minor revisions.

Major strengths
- Methodological advance with clear payoff: A GPU-ready near-atomistic model enabling simulations (condensates and long fibers) previously out of reach at this resolution.
- Substantial validation: Captures key experimental trends in compaction vs linker length and reproduces structural descriptors consistent with cryo-EM/cryo-ET/X-ray benchmarks.
- Mechanistic clarity: The 10N vs 10N+5 comparison yields an interpretable picture (stacking vs frustration; tail sequestration vs exposure; consequences for valency/connectivity).
- Phase behavior addressed directly: Direct coexistence simulations elevate the work beyond qualitative clustering narratives.
- Long-fiber acetylation analysis: Pattern-dependent folding ("hub/spacer" behavior of unmodified vs acetylated stretches) is interesting and testable.

Target issues to refine (minor revisions)
1. Tone and claim strength. Please soften statements such as "for the first time," "uncovers the mechanism," "reduces free energy," and similar superlatives unless backed by direct quantitative evidence (e.g., free-energy/entropy decomposition). Several claims read stronger than necessary.
2. Scope statements up front (medium, missing components, interpretation). The model uses implicit solvent/ions and Debye–Hückel screening; please make this explicit early and reiterate in the Discussion. Also state clearly what is not included (e.g., non-chromatin crowders/clients/readers, nuclear architecture). This will prevent over-interpretation.
3. Divalent ions (Mg2+) and ion-specific effects. Because chromatin condensates in vitro/in vivo often involve some Mg2+, please explicitly discuss that Debye–Hückel/implicit-ion electrostatics cannot capture multivalent-ion correlations/bridging/specific binding. Frame reported phase boundaries and material-property trends as conditional on an effective mean-field electrolyte (largely monovalent screening). Please also clarify the experimental ionic conditions used for comparisons (Mg2+ present vs chelated) and, if feasible, add a brief sensitivity/discussion showing how stronger/weaker screening shifts key conclusions.
4. Equilibrium vs non-equilibrium framing. Direct coexistence is an equilibrium approach; please add one concise statement clarifying that the work targets effective equilibrium thermodynamics and does not model active ATP-driven processes (remodeling, transcriptional machinery, loop extrusion), except conceptually.
5. Time-scale interpretation and mass scaling. Because coarse-graining and mass-scaling are used, please clarify what reported time units represent and avoid implying quantitative mapping to experimental kinetics unless a calibration is provided.
6. Criticality language. If the manuscript uses terms like "near-critical," please provide an operational definition and supporting diagnostics (finite-size considerations, susceptibility/correlation length proxies, etc.). Otherwise rephrase more conservatively (e.g., "near the phase boundary / weak phase separation").
7. Fit vs prediction clarity. Please clearly separate which aspects were tuned to match experiment (e.g., compaction trends) from out-of-sample predictions (e.g., coexistence densities, inferred material-property differences, acetylation-pattern effects).
8. Definitions and robustness checks. Provide one unambiguous definition for contacts/valency/network connectivity (including distance cutoffs) and the precise "energy-weighted contact" measure. Please include cutoff sensitivity and uncertainty estimates (block averaging and/or independent repeats) for central claims.
9. Direct-coexistence "slab" description and finite-size convergence. The elongated box dimensions can be misread as the box being a slab. Please clarify that the dense phase forms a slab-like domain within an elongated periodic box, producing two interfaces under PBC. In addition, please provide block-averaged density profiles along the long axis (plateaus, interface width, time stability) and, if feasible, a modest size-sensitivity check (e.g., cross-section or number of arrays) to address capillary-wave/interface finite-size effects.
10. Generality of the coexistence system. Please keep the narrative explicit that the direct-coexistence phase study uses many copies of short tetranucleosome (4-nucleosome) arrays. A brief note on how conclusions may extend (or not) to longer fibers/heterogeneous linkers would help avoid overgeneralization.
11. Literature scope (only if broad "genome organization" language remains). If the manuscript retains broad claims about genome-scale organization, please consider citing representative mesoscale/ensemble chromatin models to contextualize regimes beyond near-atomistic fibers/condensates (optional; helps align scope).

In summary, the manuscript is strong and the core contributions are compelling. The revisions requested above are primarily aimed at tightening claim scope, clarifying interpretation (especially regarding implicit electrostatics and Mg2+), and documenting robustness for the coexistence setup and analysis definitions.

Sincerely,

(Remarks on code availability)

Version 1:

Reviewer comments:

Reviewer #2

(Remarks to the Author)
The authors have adequately addressed my questions. I appreciate their detailed efforts and look forward to the exciting results from their future studies, such as those on breathing nucleosomes and sequence specificity.

(Remarks on code availability)
The authors have addressed my previous comments.

Reviewer #3

(Remarks to the Author)
I thank the authors for their careful and constructive revision. In my view, the revised manuscript satisfactorily addresses the concerns raised in my previous review. The authors have appropriately softened overstrong claims, clarified the model scope and limitations, including implicit Debye-Hückel electrostatics and the absence of divalent-ion-specific effects, active processes, non-chromatin crowders, and nuclear architecture. They have also clarified the interpretation of simulation times, distinguished fitted observables from model predictions, and added useful definitions and robustness checks for contact, valency, network, direct-coexistence, and acetylation analyses.

I am particularly satisfied with the added discussion of the direct-coexistence geometry and finite-size sensitivity, as well as the clearer statement that the phase-separation simulations are performed on tetranucleosome arrays and should not be overgeneralized quantitatively to more complex chromatin substrates.

I have no remaining substantive concerns. I recommend acceptance.

(Remarks on code availability)

Open Access This Peer Review File is licensed under a Creative Commons Attribution 4.0 International License, which permits use, sharing, adaptation, distribution and reproduction in any medium or format, as long as you give appropriate credit to the original author(s) and the source, provide a link to the Creative Commons license, and indicate if changes were made.
In cases where reviewers are anonymous, credit should be given to 'Anonymous Referee' and the source.
The images or other third party material in this Peer Review File are included in the article's Creative Commons license, unless indicated otherwise in a credit line to the material. If material is not included in the article's Creative Commons license and your intended use is not permitted by statutory regulation or exceeds the permitted use, you will need to obtain permission directly from the copyright holder.
To view a copy of this license, visit https://creativecommons.org/licenses/by/4.0/

Point-by-point replies to reviewers

Reviewer #1

This MD study represents a major advance in the simulation of larger system of nucleosome fibers with residue resolution. A crucial step to reach relevant system sizes is to replace the rigid basepair representation of the DNA model by a fully particle based description. This leads to a speed-up of about one order of magnitude, for the first time allowing to connect residue level details to reasonably large scales. The model has been reparametrized compared to the slower model and is highly predictive (e.g. sedimentation coefficients, condensate formation). This leads to exciting insights and promises to be of great importance also for future studies. For instance, Fig. 3 reports how the contacts of histone tails to other nucleosomes depend on the DNA linker length. Access to this kind of information is crucial as it allows to predict and interpret a wide range of physical properties and associated biological functions of chromatin. The section on histone tail acetylations is a concrete proof of this, as it is demonstrates that acetylations which have a strong effect on interactions in the model correspond to residues that also play an important role as acetylation marks in the biological system. I strongly recommend publication of this well-written manuscript after the following points (all minor) have been considered.

We thank the reviewer for their positive assessment of the manuscript and for recognizing the methodological advance and biological relevance of the residue-level chromatin simulations. We have revised the manuscript to address each of the minor points raised.

1. I noticed that figures in the main text are not always referred to. For instance, Fig. 3c and d do not appear to have been mentioned in the main text. In my opinion, any part of a figure which is not used in the main text belongs to supplementary material. Please check throughout the manuscript.

We thank the reviewer for identifying this issue with out manuscript. We have checked the main figures throughout. We have revised the text so that all Fig. 3 panels are explicitly discussed in the Results.

Changes made:

- Fig. 3c is now cited where the residue-level DNA-protein and protein-protein inter-nucleosome contact analysis is introduced.
- Fig. 3d is now cited when discussing the surface localization of the H4 and H2A N-terminal tails.
- We checked the remaining main figures for comparable orphaned panels.

2. When arriving at the conclusion and reading about the differences with respect to histone tail conformations between 10N and 10N+5 linkers, I had the impression, that I had read about this point already several times before in this manuscript. Please check whether this is really the case and if so, please try to shorten or remove repetitions. But please leave the text in the discussion untouched, as it is well written and provides an excellent summary.

We agree that parts of the Results repeated the mechanistic interpretation later summarized in the Discussion. Therefore, with the changes listed below, we streamlined the Results text while preserving the Discussion section, which the reviewer found useful.

Changes made:
- We removed or shortened repeated statements about H4/H2A tail exposure and linker-length-dependent histone-tail behavior in the Results.
- Retained the Discussion-level synthesis of the 10N versus 10N+5 mechanism.
- Softened some repeated mechanistic language to avoid overstatement and improve flow.

3. line 112: when referring to near-atomistic coarse grained models, please also cite work by Giovanni Brandani & Shoji Takada [I am not one of the authors].

We have added a new citation to the Introduction where near-atomistic coarse-grained models are introduced. https://doi.org/10.1093/nar/gkaf189

4. typos: line 182: "represented represented"; line 604: earleir
line 604: do you really mean Fig. 5a?
FigS1: figure has parts a and b but legend does not refer to this.

We have corrected the following typographical errors and the incorrect figure reference.

Changes made:
- Corrected "represented represented" to "represented".
- Corrected "earleir" and other minor spelling/grammar errors identified during the revision.
- Corrected the erroneous Fig. 5 panel reference.
- Fig. S1 caption now separately describes the side-by-side atomistic/coarse-grained renderings and the cut-away bead-and-bond views.

5. Finally, this new approach allows for many exciting projects in the future. My "wish list" for future projects would include that the authors consider pulling on single chromatin fibers, isolated or immersed in a condensate, and to figure out the different contributions (DNA elasticity, histone tails …) to the force-extension curves for different linker lengths. Also I would consider it highly interesting if the authors manage to allow for more extended nucleosome breathing in their model (not just 10 bps from each end) and then study how the breathing of individual nucleosomes is affected by being part of a chromatin fiber/condensate.

We thank the reviewer for these comments and suggestions, we completely agree that these are important future applications of OpenCGChromatin and are actually actively working on this, so hopefully we will be able to report on these studies soon.

Reviewer #2

This manuscript presents an exciting advance in the field of chromatin modeling and simulation by introducing a GPU-accelerated, OpenMM-based, near-atomistic (residue-resolution) chromatin model that was reparameterized from the authors' earlier work implemented in LAMMPS on CPUs [25]. The model has been applied to simulate a large chromatin system comprising 108 nucleosomes and to study liquid-liquid phase separation of tetranucleosomes with different linker lengths, presenting a compelling demonstration of the model's application to large chromatin systems with atomistic accuracy and providing insights into the role of disordered chromatin regions in phase separation.

We thank the reviewer for recognizing the advance represented by OpenCGChromatin and for the detailed suggestions regarding validation, parameterization, and model scope. We have revised both the main text and Supplementary Information to make the modeling choices, validation targets, and limitations clearer.

I have several suggestions and questions regarding the validation and parameterization of this model.

1. The authors imposed 10 base pairs of nucleosomal DNA unwrapping in their model. This small degree of nucleosome unwrapping incurs close to zero free energy penalty (see Forties et al., 2011, Nucleic Acids Res) and therefore reflects the "unbreathing" mode described in the authors' earlier paper [25]. More rationale needs to be provided for this choice, as [25] determines that nucleosome breathing is critical for nucleosome interactions, especially in phase separation.

We have expanded the rationale for this modeling choice. The revised text clarifies that the central 127 bp of nucleosomal DNA are included in the core elastic network to preserve nucleosome spacing during chromatin-fiber simulations, while the terminal 10 bp on each flank remain flexible to allow local entry/exit breathing. For synthetic chromatin fibers assembled on strong nucleosome positioning sequences, we have found in our previous work using cryo-ET to visualise such chromatin inside condensates that breathing is negligible. Therefore, we have opted for this pragmatic stabilization scheme for nucleosomes on strong positioning sequences. Current new work from our group focuses on incorporating nucleosome unwrapping for naturally-occurring DNA sequences.

Changes made:
- Main text now states that the central 127 bp are included in the elastic network while terminal 10 bp remain flexible.
- SI now explains that this choice is motivated by the Luger nucleosome structure, where the terminal 10 bp segments are essentially straight, and by evidence that spontaneous site exposure/accessibility is strongest near entry/exit DNA.
- Added Anderson/Widom 2000 citation for entry/exit accessibility.
- Added explicit caveat that this preserves nucleosome spacing and prevents passive repositioning/disassembly during fiber simulations, while retaining local breathing at entry/exit sites.

2. The manuscript shows excellent reproduction of the chromatin compaction difference between 10N and 10N+5 linker DNA, with numbers almost quantitatively reproducing the experimental results. However, the authors seem to suggest this arises from their incorporation of the CGeNArate model, a sequence-dependent DNA model. The differences in chromatin structure at different linker DNA lengths more likely arise from geometry, as analyzed in this paper, which appears to somewhat contradict the sequence-specific effect. Can the authors reconcile these observations?

Thanks for highlighting a point that needed clarification, we have revised the text to separate these concepts more clearly. The 10N versus 10N+5 compaction difference in the present validation set primarily reflects linker-DNA geometry, helical phasing, and mechanical constraints. CGeNArate is an important technical advance because it provides a particle-based, OpenMM-compatible, sequence-dependent DNA model with realistic DNA mechanics, but the specific linker-length periodicity studied here is not claimed to arise from sequence variation.

Changes made:
- Clarified the model description so that CGeNArate is presented as the DNA mechanical/sequence-dependent representation, not as the direct cause of the 10N versus 10N+5 periodicity in regular 601 arrays.
- Added fit-versus-prediction language stating that parameters were tuned against sedimentation trends for 25, 30, and 58 bp regular arrays, while other linker lengths, cryo-ET comparisons, condensate properties, and acetylation effects are predictions.

3. Similarly, in Figure S3, the authors show that their original LAMMPS model is unable to reproduce the different chromatin structures at 10N and 10N+5 linker DNA, but was this performed in unbreathing mode? If so, would allowing more nucleosomal DNA unwrapping enable the model to reproduce the trend?

We have expanded the SI discussion of the previous LAMMPS model and its limitations. The revised SI explains the trade-off encountered in the previous parameterization: stronger non-bonded protein-DNA interactions could stabilize nucleosomes when DNA was flexible, but in chromatin-fiber simulations these interactions did not robustly preserve nucleosome spacing on accessible simulation timescales. Alternatively, fully restraining the nucleosomal DNA preserved spacing but did not reproduce the experimental linker-length compaction trend. OpenCGChromatin addresses this trade-off by using weaker generic protein-DNA attractions while preserving nucleosome registration through the core ENM.

Changes made:
- Expanded SI Section 2.1 comparison to the previous LAMMPS model.
- Clarified that the previous high-resolution model did not capture the 10N versus 10N+5 sedimentation periodicity under the tested conditions.
- Added explanation that OpenCGChromatin uses a different stabilization strategy: central 127 bp in the ENM, terminal 10 bp flexible, and rebalanced protein-DNA interactions.

4. Similarly, would testing sequence-dependent chromatin properties provide a more direct validation of the model’s advancement in incorporating DNA sequence specificity? For example,

Cui et al. (PNAS, 2000) measured nucleosome pulling forces on chicken erythrocyte chromatin fiber, while Kruithof et al. (Nat. Struct. Mol. Biol., 2009) measured forces on pulling 601-sequence chromatin. Validation of these or other sequence-dependent chromatin properties by this model would strengthen its applicability for studying DNA sequence specificity.

We agree that direct validation against sequence-dependent chromatin mechanics would be valuable. However, these experiments are beyond the scope of the current manuscript, which focuses on establishing the model, validating linker-length-dependent compaction and structural trends against experiments with well defined DNA sequences, and demonstrating condensate/long-fiber applications. We are currently actively working on simulations will other DNA sequence and hope to report on these soon..

Changes made:
- Explicitly noted that such studies could separate contributions from DNA elasticity, nucleosome geometry, and tail-mediated interactions.

5. One of the highlights is that the model uses a relatively small number of parameters (Table S1) while being able to reproduce a large number of structural properties of chromatin. To achieve this, the authors used a grid search to find the optimal parameters. Would it be beneficial to also show results at 5 mM NaCl, the lowest salt concentration, and demonstrate that the model can fully reproduce the fully expanded chromatin at very low salt concentration?

We thank the reviewer for this suggestion. We have added 5 mM simulations to the Supplementary Information. These simulations show broad qualitative agreement but larger deviations from experiment at very low salt and longer linker lengths. We discuss this as consistent with the expected limitations of Debye-Huckel electrostatics at long Debye lengths, where mean-field screening can overestimate long-range DNA–DNA repulsion and neglect ion-correlation effects.

Changes made:
- Added new SI Fig. S10 showing sedimentation coefficients at 5, 60, and 150 mM.
- Added main-text discussion noting that 5 mM simulations are more extended than experiment, especially for longer linkers.
- Added explanation that this behavior is consistent with limitations of Debye-Huckel screening at low ionic strength.
- SI now states typical model use is lambda_D = 0.8-2.0 nm and that agreement decreases at the extremely low-salt 5 mM condition, where lambda_D is approximately 4.36 nm.

6. In Figure 3, the authors could consider showing intra-nucleosome versus inter-nucleosome contact frequencies. This would strengthen the authors' later analyses of those interactions at different linker DNA lengths.

We have added an SI control separating parental/intra-nucleosomal histone-tail contacts from inter-nucleosomal contacts. This confirms that the linker-length dependence emphasized in the main text is driven by inter-nucleosomal contacts, while parental contacts are comparatively insensitive to linker length.

Changes made:
- Added new SI Fig. S12 showing intra-nucleosomal/parental histone-tail contact frequencies.
- Added main-text sentence explicitly stating that inter- versus intra-nucleosomal decomposition confirms the linker-length dependence is driven by inter-nucleosomal contacts.

Minor:
1. Can the authors explain the overall smaller sedimentation coefficient (more expanded chromatin) from simulations at 60 mM compared to the experimental results?
We have expanded the discussion of this discrepancy. The revised manuscript explains that the deviation may arise from limitations in linker-DNA torsion/bending or overestimation of DNA-DNA electrostatic repulsion under symmetric Debye-Huckel screening. The discrepancy grows at lower salt and longer linkers, consistent with the expected limitations of the electrostatic treatment.

Changes made:
- Added main-text explanation of the 60 mM discrepancy.
- Added low-salt 5 mM simulations in SI Fig. S10 to probe this behavior further.
- Added broader Discussion limitations on implicit electrostatics and ion-specific effects.

2. The simulations reported in Figure 7 could be extended so that larger portions of the simulation trajectories are included in the analysis.
We extended the analysis window for the 108-nucleosome acetylation simulations.

Changes made:
- Long-fiber contact and crowding-density analysis now uses the final 40 microseconds rather than the final 25 microseconds.
- Main Methods now states that the last 40 microseconds of each 100 microsecond simulation were used for contact and crowding-density analysis.

3. The final 16x benchmark report on the 12-nucleosome fiber in Figure 1 is noteworthy and is often overlooked within the computational community. The authors could also report computer memory usage, which would provide helpful guidance for selecting appropriate GPU types for these simulations.

We have added GPU memory footprints to the SI performance benchmark table.

Changes made:
- SI Table S3 now reports approximate process-resident GPU memory usage measured with nvidia-smi during production.
- Main text now notes that the low VRAM usage enables multiple independent simulations to run concurrently with NVIDIA MPS.

4. Some typos, such as IIA: "which represented represented histones at residue resolution and DNA at the base-pair-step level using ellipsoids"
We corrected the identified typos and performed an additional copy-edit pass.

Changes made:

- Corrected "represented represented", "Fluoresence", "crystalography", and other typographical issues.

Reviewer #2 (Remarks on code availability):

The authors have provided the necessary codes to reproduce the results presented in this manuscript. I did not run through their examples directly. I suggest that the authors could include more detailed instructions on how to execute these codes, specifying the input and expected output for each code. Additionally, it would be helpful if the codes were organized according to the results reported in the manuscript.

We have clarified code availability. The OpenCGChromatin simulation package itself is available through the existing GitHub repository. For the paper-specific materials, we will provide a companion repository containing simulation setup scripts and selected analysis workflows supporting the main quantitative results, including contact-analysis workflows. This organization will make clear which workflows correspond to the manuscript analyses while avoiding unnecessary inclusion of every plotting or exploratory analysis script.

Changes made:
- Added Code Availability statement for the OpenCGChromatin package.
- Added planned companion repository: https://github.com/CollepardoLab/Russell_2026
- The companion repository is described as containing paper-specific simulation setup scripts and selected analysis workflows supporting the main quantitative results, including contact-analysis workflows.

Reviewer #3

Dear Editor and Authors,

This manuscript presents OpenCGChromatin, a near-atomistic coarse-grained chromatin framework that substantially extends chemically specific simulations into the regime of hundreds of nucleosomes and gene-length DNA, while retaining residue/nucleotide-level interpretability. The work is timely and impactful: it combines (i) strong validation against established linker-length–dependent compaction/structural benchmarks, (ii) direct-coexistence simulations to interrogate chromatin phase behavior, and (iii) a clear mechanistic narrative linking linker geometry to nucleosome stacking frustration, histone-tail exposure, multivalency/network connectivity, and condensate formation. Overall, I find the manuscript strong and well executed.

Recommendation: Accept pending minor revisions.

Major strengths
- Methodological advance with clear payoff: A GPU-ready near-atomistic model enabling simulations (condensates and long fibers) previously out of reach at this resolution.
- Substantial validation: Captures key experimental trends in compaction vs linker length and reproduces structural descriptors consistent with cryo-EM/cryo-ET/X-ray benchmarks.
- Mechanistic clarity: The 10N vs 10N+5 comparison yields an interpretable picture (stacking vs frustration; tail sequestration vs exposure; consequences for valency/connectivity).
- Phase behavior addressed directly: Direct coexistence simulations elevate the work beyond qualitative clustering narratives.
- Long-fiber acetylation analysis: Pattern-dependent folding (“hub/spacer” behavior of unmodified vs acetylated stretches) is interesting and testable.

We thank the reviewer for their supportive comments on our work and highlighting its major strengths. It made us happy to read this.

Target issues to refine (minor revisions)
1. Tone and claim strength. Please soften statements such as “for the first time,” “uncovers the mechanism,” “reduces free energy,” and similar superlatives unless backed by direct quantitative evidence (e.g., free-energy/entropy decomposition). Several claims read stronger than necessary.

We have softened several claims throughout the manuscript to avoid overstatement. We removed or revised superlative language and avoided unsupported free-energy/entropy claims.

Changes made:
- Replaced "breakthrough" with "advance".
- Removed "for the first time" from the Introduction.
- Replaced/softened "uncovers" with "elucidates" or more conservative phrasing.
- Removed a strong enthalpy/entropy claim from the Introduction.
- Removed the earlier speculative paragraph about adaptive expansion/free-energy compensation from the Discussion.
- Rephrased several broad genome-organization claims as chromatin-structure/phase-behavior claims.

2. Scope statements up front (medium, missing components, interpretation). The model uses implicit solvent/ions and Debye–Hückel screening; please make this explicit early and reiterate in the Discussion. Also state clearly what is not included (e.g., non-chromatin crowders/clients/readers, nuclear architecture). This will prevent over-interpretation.

We have made the scope of the model explicit early in the manuscript and reiterated it in the Discussion.

Changes made:
- Main model description now states that solvent and mobile ions are treated implicitly via Debye-Huckel electrostatic screening.
- Discussion now states that the model does not include non-chromatin macromolecular crowders, chromatin-binding proteins such as readers/writers/remodelers, or nuclear architectural constraints.
- Discussion now frames the results as isolating intrinsic physicochemical behavior of chromatin within this model scope.

3. Divalent ions (Mg^{2+}) and ion-specific effects. Because chromatin condensates in vitro/in vivo often involve some Mg^{2+}, please explicitly discuss that Debye–Hückel/implicit-ion electrostatics cannot capture multivalent-ion correlations/bridging/specific binding. Frame reported phase boundaries and material-property trends as conditional on an effective mean-field electrolyte (largely monovalent screening). Please also clarify the experimental ionic conditions used for comparisons (Mg^{2+} present vs chelated) and, if feasible, add a brief sensitivity/discussion showing how stronger/weaker screening shifts key conclusions.

We have added an explicit Discussion paragraph on the limitations of Debye-Huckel electrostatics for divalent ions and ion-specific effects.

Changes made:
- Added text explaining that Mg^{2+} can mediate ion bridging, correlation-driven attraction, and specific binding to nucleosomal DNA.
- Stated that these effects are not captured by a mean-field monovalent screening model.
- Framed reported phase boundaries and material properties as conditional on an effective monovalent electrostatic environment.
- Clarified that the experimental comparisons use defined monovalent salt conditions (100-150 mM NaCl/KOAc)
- Added/expanded low-salt discussion around Debye-Huckel limitations.

4. Equilibrium vs non-equilibrium framing. Direct coexistence is an equilibrium approach; please add one concise statement clarifying that the work targets effective equilibrium thermodynamics and does not model active ATP-driven processes (remodeling, transcriptional machinery, loop extrusion), except conceptually.

We added this clarification directly in the Results section that introduces the direct-coexistence analysis.

Changes made:

- Main text now states that the direct coexistence approach targets effective equilibrium thermodynamics.
- It explicitly states that active ATP-driven processes such as chromatin remodeling, transcription, and loop extrusion are not modeled.

5. Time-scale interpretation and mass scaling. Because coarse-graining and mass-scaling are used, please clarify what reported time units represent and avoid implying quantitative mapping to experimental kinetics unless a calibration is provided.

We added a Methods statement clarifying the interpretation of simulation time.

Changes made:
- Methods now state that because coarse-graining and mass scaling alter effective friction and inertia, reported times correspond to simulation time and should not be directly mapped to experimental kinetics without independent calibration.
- The same paragraph notes that equilibrium/time-averaged properties are unaffected by mass scaling because particle masses cancel in the Boltzmann factor.

6. Criticality language. If the manuscript uses terms like “near-critical,” please provide an operational definition and supporting diagnostics (finite-size considerations, susceptibility/correlation length proxies, etc.). Otherwise rephrase more conservatively (e.g., “near the phase boundary / weak phase separation”).

We rephrased the relevant language conservatively rather than introducing a formal criticality analysis.

Changes made:
- Removed/avoided unsupported "near-critical" framing.
- Used "near the phase boundary", "weak phase separation", or more direct descriptions of weaker condensate stability where appropriate.

7. Fit vs prediction clarity. Please clearly separate which aspects were tuned to match experiment (e.g., compaction trends) from out-of-sample predictions (e.g., coexistence densities, inferred material-property differences, acetylation-pattern effects).

We have added explicit fit-versus-prediction language to the model-validation section.

Changes made:
- Main text now states that parameters were tuned against linker-length-dependent compaction trends from sedimentation experiments for regular reconstituted arrays with linker lengths 25, 30, and 58 bp.
- It then states that all other results reported in the work - including sedimentation coefficients for other linker lengths, cryo-ET structural comparisons, condensate properties, and acetylation effects - are model predictions.

8. Definitions and robustness checks. Provide one unambiguous definition for contacts/valency/network connectivity (including distance cutoffs) and the precise "energy-weighted contact" measure. Please include cutoff sensitivity and uncertainty estimates (block averaging and/or independent repeats) for central claims.

We substantially expanded the Methods and SI definitions and added robustness/uncertainty reporting.

Changes made:
- Main Methods now defines residue-residue contacts as r_c = 1/2(sigma_i + sigma_j) + 0.25 nm.
- Main Methods defines non-parental/inter-nucleosomal contacts as contacts between residues belonging to different nucleosomes, with nucleosomal DNA and both linkers considered part of each nucleosome.
- Main Methods defines nucleosome-nucleosome contacts using a 13 nm center-of-geometry cutoff and orientation classification.
- Main Methods defines energy-weighted contacts as pairwise Ashbaugh-Hatch plus Debye-Huckel energy evaluated at instantaneous separation.
- For Fig. 8c, Methods now states that the WT versus acetylated lysine analysis uses a 1.6 nm (2 lambda_D) cutoff before energy weighting.
- SI gives mathematical definitions of residue contacts, nucleosome contacts, orientation categories, energy weighting, radius of gyration, structural clustering, network connectivity, and valency.
- Added SI Fig. S11 for nucleosome-contact cutoff robustness (12, 13, and 14 nm).
- Added SI Fig. S13 for residue-level tail-contact cutoff robustness.
- Added SI Fig. S17 for acetylation analysis controls: close-contact energy-weighted contributions and raw contact frequencies at close-contact and 1.6 nm Debye-range cutoffs.
- Added global block-averaging statement and figure-caption uncertainty statements: means are computed over five non-overlapping trajectory blocks; error bars/shaded bands show SD across block means.

9. Direct-coexistence "slab" description and finite-size convergence. The elongated box dimensions can be misread as the box being a slab. Please clarify that the dense phase forms a slab-like domain within an elongated periodic box, producing two interfaces under PBC. In addition, please provide block-averaged density profiles along the long axis (plateaus, interface width, time stability) and, if feasible, a modest size-sensitivity check (e.g., cross-section or number of arrays) to address capillary-wave/interface finite-size effects.

We have clarified the direct-coexistence geometry and added additional robustness analyses.

Changes made:
- Main Methods now says direct coexistence simulations place 81 arrays in an elongated periodic box where the dense phase forms a slab-like domain spanning the short cross-section and producing two planar interfaces along the long axis under periodic boundary conditions.
- Main Fig. 4 caption now reports that density profiles are block-averaged and uses shaded bands for SD across five block means.
- SI now gives the density-profile and interface-fitting procedure, including local hyperbolic-tangent fits to the two interfaces, Gaussian smoothing only to locate interfaces, fitting the

unsmoothed profile in local windows, and excluding 1.5|w| around interfaces for plateau densities.
- Added SI Table S6 with block-by-block dense-phase density, dilute-phase density, and interfacial width for 25 and 30 bp coexistence simulations, including mean +/- SD across five blocks.
- Added SI Fig. S16 testing robustness to modest changes in direct-coexistence box cross-section; both density profiles and downstream structural clustering are stable under the tested cross-section changes.

10. Generality of the coexistence system. Please keep the narrative explicit that the direct-coexistence phase study uses many copies of short tetranucleosome (4-nucleosome) arrays. A brief note on how conclusions may extend (or not) to longer fibers/heterogeneous linkers would help avoid overgeneralization.

We added a scope statement to avoid overgeneralizing the tetranucleosome direct-coexistence results.

Changes made:
- Main text now explicitly notes that the direct coexistence phase study uses short tetranucleosome arrays with uniform linker lengths.
- Added text stating that while the core mechanism - linker-DNA-imposed nucleosome orientations controlling tail accessibility and multivalent engagement - is expected to operate in longer fibers and heterogeneous linker sequences, quantitative phase boundaries and material properties may differ for more complex chromatin substrates.

11. Literature scope (only if broad "genome organization" language remains). If the manuscript retains broad claims about genome-scale organization, please consider citing representative mesoscale/ensemble chromatin models to contextualize regimes beyond near-atomistic fibers/condensates (optional; helps align scope).

We have softened broad genome-organization claims

Changes made:
- Rephrased several broad claims from genome organization to chromatin structure, chromatin phase behavior, or intrinsic chromatin physicochemical behavior.
- Added model-scope language clarifying that nuclear architecture and non-chromatin factors are not included.
